# Event-related potential datasets based on a three-stimulus paradigm

**DOI:** 10.1186/2047-217X-3-35

**Published:** 2014-12-12

**Authors:** Lukas Vareka, Petr Bruha, Roman Moucek

**Affiliations:** Department of Computer Science and Engineering, Faculty of Applied Sciences, University of West Bohemia, Univerzitní 8, 306 14 Plzeň, Czech Republic

**Keywords:** Event-related potentials, P300, Three-stimulus paradigm, Visual stimulation, LED

## Abstract

**Background:**

The event-related potentials technique is widely used in cognitive neuroscience research. The P300 waveform has been explored in many research articles because of its wide applications, such as lie detection or brain-computer interfaces (BCI). However, very few datasets are publicly available. Therefore, most researchers use only their private datasets for their analysis. This leads to minimally comparable results, particularly in brain-computer research interfaces.

Here we present electroencephalography/event-related potentials (EEG/ERP) data. The data were obtained from 20 healthy subjects and was acquired using an odd-ball hardware stimulator. The visual stimulation was based on a three-stimulus paradigm and included target, non-target and distracter stimuli. The data and collected metadata are shared in the EEG/ERP Portal.

**Findings:**

The paper also describes the process and validation results of the presented data. The data were validated using two different methods. The first method evaluated the data by measuring the percentage of artifacts. The second method tested if the expectation of the experimental results was fulfilled (i.e., if the target trials contained the P300 component). The validation proved that most datasets were suitable for subsequent analysis.

**Conclusions:**

The presented datasets together with their metadata provide researchers with an opportunity to study the P300 component from different perspectives. Furthermore, they can be used for BCI research.

## Data description

### Purpose of the study

In recent decades, research into event-related potentials (ERP) using a classic odd-ball paradigm has become very popular. However, studies on the neural substrates of the P300 and other ERP components are still lacking. In [1], the authors propose to use a three-stimulus paradigm to explore the P300 component in more detail. The purpose of this study was to make three-stimulus paradigm EEG/ERP datasets freely available to the neuroinformatics community. To the authors’s knowledge, no three-stimulus paradigm datasets have been published.

The experiments were based on visual stimulation, aimed at healthy subjects; the resulting datasets are stored in the EEG/ERP Portal [2] and in the GigaScience database, GigaDB ([3]).

### Experimental design

#### Recording software

The BrainVision Recorder 1.2 was used [4] for recording and storing the EEG/ERP data in the BrainVision format. The Recorder was initialized using the following parameters:

- the sampling rate was set to 1 kHz
- the resolution was set to 0.1 *μ*V
- the recording low-pass filter was set with the cut-off frequency of 250 Hz

The impedance threshold was set to 10 *kΩ*, and the real impedances for each experiment are stored as vhdr files.

#### Stimulator

**Figure 1:**
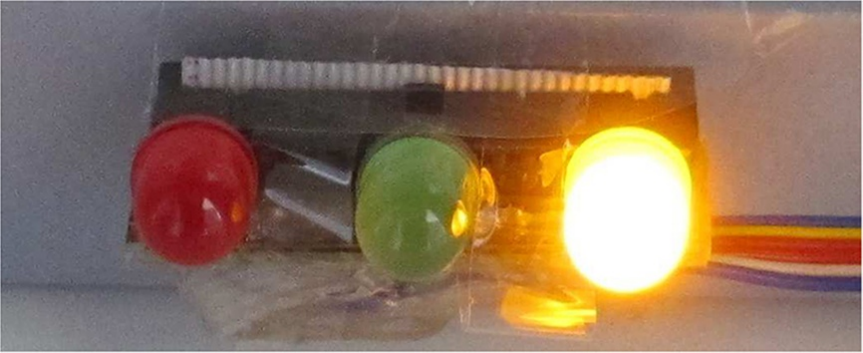
**Stimulator with flashing diodes.**

The stimulator [5] includes three high-power Light-Emitting Diodes (LEDs) differing in color: red, green and yellow, and this simulator has been typically used for three-stimulus paradigm [5] odd-ball experiments. Apart from traditional target and non-target stimuli, the stimulator can also randomly insert distractor stimuli. Figure 1 shows the LED module with the yellow diode flashing.

#### Stimulation protocol

The stimulator described above was used in the stimulation protocol. In our experiments, the stimulator settings were used as follows: each diode flashed once a second and each flash took 500 ms. The probabilities of the red, green and yellow diodes flashing were 83%, 13.5% and 3.5%, respectively. Between two occurrences of target stimulus (green diodes flashing), at least one non-target stimulus appeared. Otherwise, the order of stimuli was completely random.

The participants were sitting 1 m from the stimulator for 20 minutes. The experimental protocol was divided into three phases, each containing 30 target stimuli and each running for five minutes long. There was a short break between the phases. The participants were asked to sit comfortably, not move and to limit their eye blinking. They were instructed to pay attention to the stimulation.

#### Environment

All experiments were recorded May-July 2012 between 9 am and 5 pm. A soundproof cabin illuminated with a moderate white light was used for the experiments.

#### Participants

A group of 25 healthy volunteers participated in our experiments. However, the data from five of the volunteers were discarded because these participants were blinking excessively during the experiment. The data from the remaining 20 subjects (9 males and 11 females, university students, aged 20-26, 19 of them right-handed, half of them with corrected myopia) were stored. The informed consent was signed by all participants.

#### Procedure

**Figure 2:**
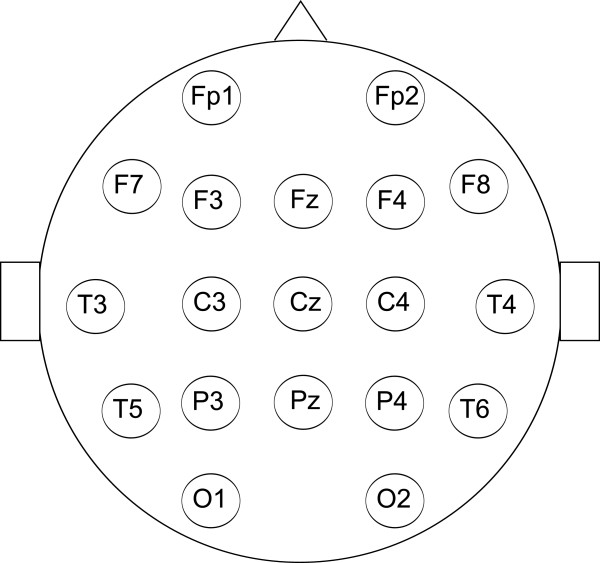
**The locations of the electrodes.**

The following experimental procedure was applied:

- Each participant was acquainted with the course of the experiment and answered questions concerning his/her health.
- Each participant was given the standard EEG cap made by Electro-Cap International. The international 10-20 system of electrode placement was used. In fact, 19 electrodes were used as depicted in Figure 2. The participant was taken to a soundproof and electrically shielded cabin, and the reference electrode was placed at the root of his/her nose.

- The participant was told to watch the stimulator, and to follow the rules described in Section “Stimulation protocol”.
- The cabin was closed and both the data recording and stimulation started.
- After the experiment had finished, the recorded data and collected metadata were uploaded to the EEG/ERP Portal.

### Data validation

#### Methods

The data were validated in two different ways:

- 1) The first test was used to check the data obtained for eye-blinking artifacts. The percentage of epochs damaged by eye blinks was estimated using visual inspection of the data for each subject separately.
- 2) The second test was used to validate the objective of the odd-ball paradigm experiments: for most participants, the target and non-target markers are expected to be associated with differently shaped ERP components, especially P2, N2, and P3 [6]. To validate this objective, dichotomous classification was used. If classification of a specific dataset yields low error rates (defined later), the objective of the odd-ball paradigm was considered to be fulfilled. Distractor stimuli that are thought to be associated with the NoGo-P300 [1] are harder to detect in the EEG signal and were thus excluded from the validation process. Furthermore, to the authors’ best knowledge, there are no datasets publicly available that contain distractor stimuli data.
  The classifier was trained on a randomly selected data subset. The training subset contained 730 ERP trials with equal numbers of targets and non-targets, whilst the trained classifier was applied to the data of individual subjects. Following this, the classifier was also tested on public data produced by another laboratory [7]. The stimulation protocol described in [7] is similar to the protocol described in this paper; it only differs in the length of inter-stimuli intervals.
  Matlab scripts available in [8] using EEGLAB and BCILAB functions [9] were used for the implementation. Both feature extraction and classification follow the Windowed Means Method proposed in [6]. This method includes feature extraction — low pass filtering and spatial filtering, and shrinkage Linear Discriminant Analysis-based machine learning. The continuous signal was split into epochs using the stimuli markers with the pre-stimulus interval for baseline correction set to 500 ms and the post-stimulus interval set to 1000 ms. As a result, the post-stimulus parts of the epochs were not overlapping. The S2 marker (the green diode flashing) corresponded to the target stimuli occurrence and the S4 marker (the red diode flashing) to the non-target stimuli occurrence. After epoch extraction, the epoch signal was band-pass-filtered with the cut-off frequencies of 0.1 Hz and 8 Hz.
  The narrow band-pass filter was used to eliminate as much undesired noise as possible for the subsequent classification. Then, each epoch was down-sampled to 100 samples. In order to extract the features, the intervals following the occurrence of stimuli were chosen as listed below:
  1. 200 ms - 250 ms
  2. 250 ms - 300 ms
  3. 300 ms - 350 ms
  4. 350 ms - 400 ms
  5. 400 ms - 450 ms
  6. 450 ms - 500 ms
  The intervals were chosen to correspond to the occurrence of ERP components that differ significantly for target and non-target stimuli [6], and for each interval, the average value for each EEG channel was calculated. These averages formed the feature vectors.

#### Results

**Figure 3:**
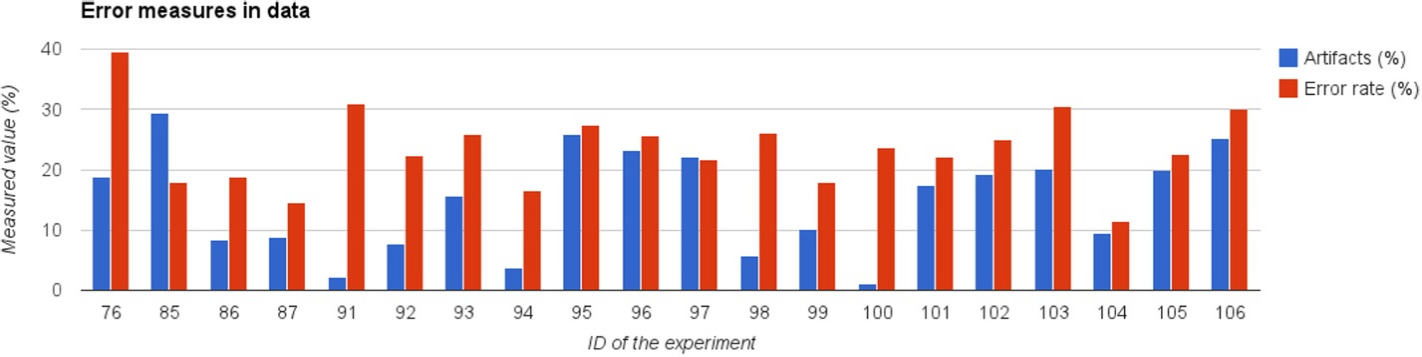
**EEG/ERP experiments — results of validation.** The percentage of eye blinking artifacts in the data and the error rate of the P300 classifier are shown.

Figure 3 shows the results obtained.

In this figure, blue bars depict the percentage of epochs damaged by eye-blinking artifacts for each participant. The participants associated with a higher artifact rate (by experience set to 15%) were unable to stop blinking excessively during the experiment. The error rates of the classifier are depicted by red bars. Let us suppose that we have *t*_*p*_ — number of correctly classified targets, *t*_*n*_ — number of correctly classified non-targets, *f*_*p*_ — number of misclassified non-targets, *f*_*n*_ — number of misclassified targets. Error rate was calculated according to Equation 1.
1

As a result, error rates indicate the extent to which the classifier was unable to separate target and non-target single trials.

Note that the classification results may differ with each run because of the indeterministic training process. For comparison, the error rates achieved for external data from three subjects [7] were 30.5%, 36.3% and 28.5%, respectively.

##### Grand averages

**Figure 4:**
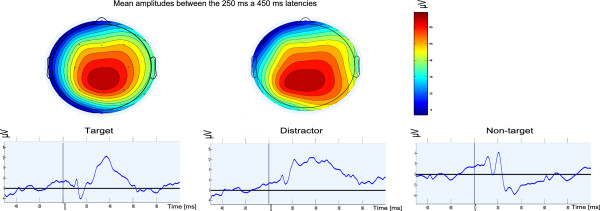
**Grand averages (Pz electrode) andscalp maps for each stimulus type.** 1808 target, 11802 non-target, and 466 distractor trials were used for averaging.

In addition to the classification method used, grand averages and related scalp maps were generated to show, how each stimulus type creates a different ERP response. For this purpose, the data from all experiments were low-pass-filtered with the cut-off frequency 30 Hz as commonly recommended for ERP experiments (e.g., in [10]). Subsequently, for each stimulus type, the trials extracted as described above were averaged. Both target and distractor trigger a response that can be seen more than 300 ms after the stimulus onset. Scalp maps depict the spatial distribution of mean values between 250 ms and 450 ms after the stimulus onset. Both markers are associated with a similar response as mentioned in [1]. Figure 4 depicts the grand averages.

## Availability and requirements

To download and analyze the data described in this article, the following projects are available:

- **Project name:** EEG/ERP Portal
  **Project home page:**http://eegdatabase.kiv.zcu.cz
  **Operating system(s):** Platform independent
  **Programming language:** Java
  **Other requirements:** tested in Internet Explorer 10, 11, Mozilla Firefox 29.0.1, Google Chrome
  **License:** GNU GPL
- **Project name:** P3-validator
  **Project home page:**https://github.com/INCF/p3-validator
  **Operating system(s):** Platform independent
  **Programming language:** Matlab
  **Other requirements:** Matlab 2010a or newer, preferably 64bit operating system
  **License:** GNU GPL

## Availability of supporting data

The data sets supporting the results of this article are available in the EEG/ERP Portal under the following URL: http://eegdatabase.kiv.zcu.cz/.

Supporting material for this paper can also be found in the GigaScience database, GigaDB ([3]).

To download the experimental data and metadata using the EEG/ERP Portal, the user must take the following steps:

- The registration form must be filled out.
- The user is logged in using his/her e-mail address and password.
- The section *Experiments* in the header of the selected page.
- The “Event-related potential datasets based on a three-stimulus-paradigm” package contains the datasets related to this article.
- The data and related metadata can be selected and confirmed after clicking on the *Download* button. By selecting “Choose all”, the user can download all the data and metadata related to the specific experiment.

## Abbreviations

BCI: Brain-computer interface
EEG: Electroencephalography
ERP: Event-related potentials
P300: Cognitive positive event-related potential component.
